# Third Apocryphal Report of the Proceedings of the Mississippi Valley Association of Dentists

**Published:** 1854-04

**Authors:** A. M. Leslie

**Affiliations:** Author of the two preceding reports and some other matters—humph—a-hem.


### ARTICLE III.

*Third Apocryphal Report of the Proceedings of the Mississippi Valley Association of Dentists.* Prepared by A. M. LESLIE, D. D. S., F. M. V. A., Author of the two preceding reports and some other matters—humph—a-hem.

MESSRS. EDITORS:—I owe your readers an apology for so long deferring my annual account of the progress of your western brotherhood. The best I can make is to state the truth, I have been very much indisposed—to the use of the pen, and am even yet an invalid. Still the advantage derived by the profession from your publishing my first report, which opened up the discussion on professional patents, followed as it was by the admission of the views of many others pro and con, by which some were taught that they could not shut out the light of the press, however easy it might be to trample on the rights of the individual or throw their weight into the balance they had formerly unmercifully kicked—prompts me to continue the reports. Fearing from remarks I have heard and some things which have been published, that my actual indifference to the base and slanderous attack made on me through the pages of the Journal, in a paper styled "A Review" of my report of the Louisville meeting, may be construed by some into a refusal of space in your Journal for my reply, I will here say that the writer of that and the cause he worked for, are welcome to the force of all the personal aspersion he has endeavored to cast on me. The time will come when he shall eat the fruit of it. Whether this shall prove figs or thistles will depend on the *facts*, not on *personal abuse;* my cause needs not the latter, and I am above it. When the harvest time arrives I shall be pleased to avail myself of the pages you so kindly offered on the occasion of the review. In the mean time, let me say to the writer of said review, that I enjoyed it much, as in addition to that which is generally derivable from his writings we got him to *express his views at length on the subject of patents.* This may be valuable for reference, as it leaves less room for ambiguity. There are some other things in which the report has induced him to give his views "*at length,*" and there are still some others that I should like to see him "delivered of" on paper, just that we might know in future where to stand, and we to find him. I am pleased to say that our editor has since that report appeared more in the press than formerly. I do not claim credit for this, oh no, it may be only a coincidence.

The title of my reports, you perceive, I have changed, simply because their nature will thus be more clearly represented to the *slow* reader. They are apocryphal only in the sense that they are not canonical, not "approved" by the council. Apocryphal because they contain words and acts *not allowed* to go into the canonical story, not because they are not true, but for the reason that they are *too* true. What, you may be ready to say, has there been more curtailing? Yes, verily, but this time moved doubtless by magnanimity, and in order that I may be relieved somewhat from the weight of favor I am under, I will at the outset, though out of place, give the apocryphal part of my report. Having before, from a sense of duty, spoken out that which the society thought derogatory to it, I could not but consider myself partial to myself if I did not uncover that which may, by some, be considered very magnanimous.

Very late on the evening of adjournment the following preamble and resolution were presented by Dr. Watt:

"Whereas we believe that all associations have a perfect right to transact their own business in their own way, therefore

"*Resolved*, That when our society, by direct vote, resolves to screen from the public eye a part of its proceedings, believing that its best interests require such course, we have a right to expect that members will comply with the spirit of such resolutions, and, at least, refrain from publishing imperfect, self-laudatory reports."

Said resolution having been seconded, and some one having expressed doubt of the necessity of the same, Dr. Leslie suggested that the mover doubtless could explain.

Dr. Watt explained by saying that reports had appeared by some one signing himself A. M. L., and said to be a member of this society, in which reports, things were exposed which the society deemed proper not to make a minute of, and members found themselves after their return home, reported to have said that which they could recollect nothing of. It was to prevent this, and have only such matters published as the society approved, that he offered the resolutions.

Dr. Allen saw just about ditto to the foregoing, and thought the adoption of the resolution necessary.

Dr. Leslie remarked, that he had very little to say on the matter, especially as he had just been informed by our host that things more worthy of discussion than this awaited us in the next room. He would, however, say that he approved in general of the sentiment, and if he voted at all he would be most likely to vote for it, it being, however, like all other generals, *subject to exceptions.* He would assure Dr. Watt that he was correct in supposing that the reports alluded to were prepared by a member of the society, and further the resolution would not necessarily have the desired effect.

The resolution passed without a dissenting vote.

On motion, it was *Resolved*, That no record be made of the resolution just adopted.

This also passed without a dissenting vote.

And now, Messrs. Editors, I am done with the non-canonical part, no one can object to the exhibition of the society's magnanimity as shown in the last resolution, much less can they to the establishment of its dignity by my voluntary publication of the first.

In my report of the former meeting I concluded by saying, that the society separated with a general expression of belief that a more useful future was open before them. The truth of this the present meeting has just proven. It has been the largest, as well, I believe, as one of the most useful ever held.

The meeting was presided over by Dr. Taft, with evident ability. Through the business committee, aided by the chairman, a system was given to the proceedings which made the discussions practical, profitable and partaken in by all the members; this brought out a variety of thought and modes which told plainly that the books are not now blindly followed.

The President opened the meeting by reading a neat and appropriate address on the "Past and Future of our Profession." This was well received.

Dr. Watt read a short but searching essay on the principles involved in professional advancement and intercourse. It produced some wincing among those who draw any nourishment through "the monster at Washington." Its publication was not very promptly desired, but we made out to secure the society's request that it be printed, which has been accomplished after four months.

Dr. Allen here took the floor to explain his position on the patent question, and his views of the society's action at a former meeting. He held forth in a loose style of animated declamation peculiar to himself, in which he repeated the oft exploded idea that patent laws are the *parents* of invention, (the almighty dollar obscures his vision so that he sees not *necessity*, the true mother of it all, known to all from Adam down to Arkwright and the inventor of the cotton gin.) And the doctor held that *nineteen-twentieths* of the profession thinks as *he* does. I can pity his ignorance, but cannot excuse his impudence, in stating anything so lacking the truth, and this to a society who has voted his course derogatory to his professional character.

All of this speech he makes out of order, and no interruption offered; and when I seek an opportunity to correct his gross misrepresentation, then it is all out of order; and when I offer a resolution which will give opportunity, it is voted down because—oh, yes! "because Allen had all the time he wants"—he tells them so, "and, they think the time can be better spent." "Oh, yes!" says the Dean, "we cannot spend time on that." And so the untruth, that only one-twentieth part of the profession disapprove of dental patents, must be allowed to pass uncontradicted in the presence of a dozen young men who are being *prepared for future usefulness.* Is it because they (the dental class) "brought down the house" with thunders of applause at the end of said speech? Teachers of youth should be wary what examples they set.

The society having resolved to proceed with the discussion of the subjects suggested by the executive committee, the first item was taken up.

"Best method of cleaning teeth and removing "green stain," deep seated, from the teeth; also, the best article to be used for that purpose."

Dr. Griffith said: He first took a sharp instrument and removed all he could with it; this he followed with slips of pumice stone; he then finished off with some polishing powder, say chalk applied by pine sticks. But one of the best things he had found for the removal of this stain, was an article, the value of which he discovered while practicing in South Carolina; it also made a most excellent dentifrice. This was the ashes of the husk of the rice plant. Its mechanical action is rapid.

Dr. Ulrey prefers the use of pumice stone and alcohol, applied with wood, as the instrument for the removal of the green stain. Has in some instances had to remove nearly all the enamel before accomplishing its perfect removal.

Dr. Allen has been in the habit of using diluted muriatic acid in cleaning the teeth, being careful to follow it immediately with an alkali, and believes, that with care, this practice may be pursued with impunity. To neutralize the acid, he uses fine Windsor soap or soda. Done neatly, the public may be kept *ignorant* of the use of the acid. Said he could use it so as not to injure the tooth, by leaving it to operate just long enough to act on the foreign substance.

Dr. Bonsal deprecated the use of acids for this purpose. His practice is to remove it with pumice stone.

Dr. Griffith disapproved of the use of acids, and thought the use of pumice should be guarded. In illustration of its injudicious use, he stated he was once consulted by a lady, a relative of Commodore Porter. She had been advised by Dr. Castle, of Baltimore, to use pumice as a dentifrice, and by its use she had, when he saw her, cut away the labial surface of the incisors, until the teeth were exceedingly sensitive.

Dr. Ayres thought it important to trace the chemical phenomena producing the stain; we might then be enabled philosophically to prepare an agent for its removal. He had heard the celebrated Professor Dudley, of Transylvania College, inculcate the doctrine, that the deposition of tartar on the teeth indicated a change in the system, which if removed, would produce secretions so altered as themselves to be the means sufficient for its removal by solution. Dr. D. taught that the earthy secretions in the kidneys and bladder were similar in chemical formation, and should be prevented by similar means. Dr. Ayres uses pumice freely on twigs of yellow poplar. He was not prepared to say whether this deposit should be attributed to an acid state of the secretions or not.

Dr. H. R. Smith uses rotten stone and pumice a good deal for the removal of the green stain. Was decidedly opposed to the use of acids on the teeth; he could not see how injury could be avoided.

Dr. Leslie thought it important in arriving at a correct opinion on this subject, that we first settle, whether the "green stain" be a deposit on the tooth or a staining of the substance of the tooth in the form of a dye. His own practice had been based on the supposition that it is a deposit, and that the hard deposit was made up of the same ingredients as the soft; that it did not enter the substance of the tooth until the surface was injured by the ordinary destructive agents. Viewing it, then, in either case, as a substance to be removed, he thought that way the best which would leave the smoothest surface, and do it the speediest. This, he believed, was to be obtained best by the edge of a sharp cutting instrumeut, followed by some polishing powder or substance; he preferred scotch stone; sometimes he entrusted the latter part of the operation to the patient—a ten minutes use of powder and brush, in most cases, being sufficient—in more important ones the burnisher adds much to the improvement of the surface. He would not attempt to decide what causes operate to produce this deposit, but thought an acid state of the fluids would not be found when the deposit was hard and black. When this and the surface of the teeth under it were partly decomposed, would consider it to indicate the action of acid. From recent observation he was inclined to believe we had too much overlooked the action of the abnormal secretions of the gums. He had enjoyed a very favorable opportunity of watching one case belonging to a class recently pointed out by an anonymous writer, viz. Scrutator. It was a very clear case of the type. When first observed, there was the yellow line of ulceration about half a line wide, pouring its secretion down on the superior incisors, which, up to this time, were white and clean, but which now rapidly assumed a yellow tint just below the ulcer. This state continued for a couple of weeks, the case being closely watched, but nothing done to arrest it. This course was adopted *that some tangible results might be arrived at.* By this time the surface attacked was a dirty brown, and the enamel decomposed across the surface of the teeth for about the width of a line and a half, being deepest in the centre of the teeth and vanishing towards the approximal surfaces—the left lateral incisor being the most injured. About the end of the third week, nothing having been done locally or constitutionally to counteract the disease, it was observed that the yellow ulcerating line of gum had changed its appearance. Its color now was natural and its acrid discharge arrested. This case evidently owed its origin to a *constitutional derangement*, ("not to candy or calomel,") the manner of removal of which is beautifully illustrative of the existence of the *vis medicatrix naturæ.* It may possibly be also illustrative of Prof. Dudley's idea, but it certainly, so far as one case goes, very fully supports the theory of "Scrutator."

The treatment of these teeth afterwards, was, in a few days, to remove with a sharp instrument, all the decomposed portion and polish them. Since then there has been no return of the disease, and *consequently* no further progress of the decay.

Regarding the suggestion thrown out by some members that it was probable that dentifrices might be prepared for use in cases having a tendency to the deposit under consideration, and which would prevent such deposit, the fluids of the mouth being either acid or alkaline, as may be proven by test paper. He would say, that he thought that the use of alkalies in dentifrices was not as guarded as it ought to be. It seemed that many overlooked the effect of such agents on the animal portion of the teeth, which of course is freely exposed to this affinity wherever decay is progressing.

It would be safest to combine in tooth-powders in general use, no more active neutralizer than creta preparata. This combined with an astringent will accomplish the chief results to be derived in a dentifrice, equally so with those that are formed of six or eight ingredients. Of course no objections are had to a little matter to throw a blush over the chalk.

Dr. Ulrey thought the green stain had an acid origin. He endeavored always to impress upon his patients the importance of the constant use of the brush and a good dentifrice. He was fully satisfied the most important period of the day for cleaning the teeth was just before retiring to sleep, as acids left in the mouth have then undisturbed possession for hours.

Dr. Griffiith said he thought the stain should be considered as a deposit, and believed it should be attributed to an acid origin.

Dr. Watt did not believe that the presence of holes in the enamel mentioned by some as accompanying this stain or deposit, proved that it had an acid origin, but only might be considered as proof of the presence of an acid combined with it.

Dr. Ayres uses an instrument when the enamel is softened. He prefers a tooth void of enamel to one with a mushy surface.

Dr. Peebles did not believe acids could be used with safety for the removal of the green stain—prefers the instrument and some polishing substance. Had found that this stain was confined to persons under mature age.

Dr. Taft thought it well to inquire into the action of the ashes of the rice plant mentioned by Dr. Griffith. If its action was so much more rapid than any other agent the doctor had met with, it might be attributable to some chemical affinity. The action of the ashes were doubtless alkaline, and if acid entered into the formation of the stain, the chemical effect was easily traceable. Then there was the mechanical action, which is said to be great, doubtless owing to the silica entering into the formation of the husk of the berry.

The origin of this deposit he thought somewhat obscure, but inclined to the belief that it results from an acid state of the fluids.

Dr. Goddard presented the following query:

"What is the best application which can be made, to remove the sensibility occasioned by the use of the file for removing decay on separating teeth?"

Dr. Goddard said he could find only two substances that could accomplish this. These were arsenic and cobalt, or free stone. He did not, however, approve of the use of these, and was anxious to know if there were any safer modes known to the members, as he had, in his own mouth, a tooth which had been cut away freely to remove caries, but there remained great sensitiveness in the dentine.

Dr. Griffith had always found entire safety in the use of arsenic when combined with charcoal. This was his mode of treating very sensitive teeth, and he could confidently recommend it.

Dr. Allen had found gum-camphor and alcohol, and also a solution of tannin in alcohol valuable in his practice when he met with very sensitive teeth. He had while east last summer presented to him by a dentist, a substance prepared and sold by Dr. Brewster, of Portsmouth, N. H., and which was designed for the obtunding the extreme sensibility of the teeth. Dr. A. here produced a small vial, duly labelled with the inventor's name, directions for use and the price. The substance was blackish-brown in color, brittle and very slight metallic taste, was in small broken fragments about half a line thick.

Dr. Peebles was in the habit of using a burnisher slightly heated in cases coming within the query of Dr. Goddard. He had found the cold burnisher sufficient in many cases. His purpose in this practice is to close up the pores of the dentine and prevent the egress of irritating fluids.

Dr. Ulrey, in speaking of the main objection to the use of arsenic for the purpose under discussion, viz. the liability of absorption, said he was applied to twelve years ago to allay sensibility in a broken tooth. He applied arsenic; the tooth continued sore for two or three months and subsided. He recently extracted the same tooth, broke it and found the tooth dead.

Dr. Smith said he believed he could let the society into a secret regarding a material for obtunding nerves. He did not know that it was exactly identical with that presented by Dr. Allen, but he thought it *very like it.* It was originally given to him by a doctor in Buffalo, as a profound and valuable secret, his friend the doctor having paid some one fifty dollars for the recipe. The mode of preparing this miraculous obtunder is to mix up some gypsum with water, add a little arsenic and color it to suit the imagination, pour it out in thin sheets, when set, break it up into fragments, it is then ready to bottle, label and sell.

Dr. Taylor makes use of a burnisher sometimes for the removal of sensibitity. He has met with great success in the use of nitrate argenti when great tenderness of the dentine existed. He had found this substance very useful when the clasps of a plate had been the cause. In one case a lady had worn some artificial teeth but two years, when the decay and sensibility was such that the teeth could not be worn. He cut away the decay and applied the silver, when the tenderness was removed and she wears the teeth. Has applied the silver four or five times, in the course of as many years, in the same case since.

